# RiceRelativesGD: a genomic database of rice relatives for rice research

**DOI:** 10.1093/database/baz110

**Published:** 2019-09-27

**Authors:** Lingfeng Mao, Meihong Chen, Qinjie Chu, Lei Jia, Most Humaira Sultana, Dongya Wu, Xiangdong Kong, Jie Qiu, Chu-Yu Ye, Qian-Hao Zhu, Xi Chen, Longjiang Fan

**Affiliations:** 1 Institute of Crop Science and Institute of Bioinformatics, Zhejiang University, Hangzhou 310058, China; 2 Zhejiang University, Hangzhou 310058, China; 3 CSIRO Agriculture and Food, GPO Box 1700, Canberra, ACT 2601, Australia

## Abstract

Rice (*Oryza sativa* L.) is one of the most important crops worldwide. Its relatives, including phylogenetically related species of rice and paddy weeds with a similar ecological niche, can provide crucial genetic resources (such as resistance to biotic and abiotic stresses and high photosynthetic efficiency) for rice research. Although many rice genomic databases have been constructed, a database providing large-scale curated genomic data from rice relatives and offering specific gene resources is still lacking. Here, we present RiceRelativesGD, a user-friendly genomic database of rice relatives. RiceRelativesGD integrates large-scale genomic resources from 2 cultivated rice and 11 rice relatives, including 208 321 specific genes and 13 643 genes related to photosynthesis and responsive to external stimuli. Diverse bioinformatics tools are embedded in the database, which allow users to search, visualize and download the information of interest. To our knowledge, this is the first genomic database providing a centralized genetic resource of rice relatives. RiceRelativesGD will serve as a significant and comprehensive knowledgebase for the rice community.

## Introduction

Crop breeding is crucial for guaranteeing food security and sustainable human population growth. Cultivated rice (*Oryza sativa* L.) is one of the most important crops worldwide and a model species for functional genomics of monocots. During the period of domestication, cultivated rice has lost many genes controlling important agronomic traits, such as resistance to abiotic and biotic stresses, which are potentially very useful for modern rice breeding (1,2). Many studies have revealed that genes regulating these traits are maintained in two closely related groups of the cultivated rice (3–5). One group includes species such as *O. rufipogon* that is phylogenetically closed to the cultivated rice (6). The other group consists of paddy weeds that have a similar ecological niche as rice and are highly competitive and readily adapt to the agroecosystem (5,7). Species of both groups are potential gene resources for modern rice molecular breeding programs aiming for improvement of agronomic traits.

With the availability of sequencing technologies, genome-based molecular approaches can increase the efficiency of rice breeding (8–12). The first rice genome was sequenced in 2002, making it the first crop genome to be deciphered (13,14). To date, dozens of *de novo* sequenced genomes of rice and its relatives have been published (7,15–20). According to the statistics from the TimeTree database (21), 13 of the 46 species in the *Oryzoideae* subfamily have already been sequenced and/or reconstructed to the pseudo-chromosome level, including seven rice relative species sharing the AA genome (16,20,22,23). Apart from the species phylogenetically close to *O. sativa*, the genome sequence of *Echinochloa crus-galli*, one of the most pervasive paddy weeds, has also been investigated (7). It demonstrated that *E. crus-galli* interacts with *O. sativa* reciprocally through allelopathy, which gives us an example of crop–weed interaction on the genomic level (7). Genomic data of rice and its relatives have provided significant and important insights into the molecular mechanisms for stress resistance, regulation of agronomic traits and interactions between cultivated rice and other plants (e.g. allelopathy) in its immediate environment (5).

To facilitate the accessibility of information from such enormous genomic data, several *Oryza* genome databases have been created to accommodate the genome data and various other types of data. The current online genomic resources of rice can be roughly divided into three categories depending on the main resources included. One is the *de novo* genome data (e.g. RAP-DB, MSU-RGAP, RIGW, RIS and RPAN) (24–27), another is rice genomic diversity data (e.g. SNP-Seek, RiceVarMap and OryzaGenome) (28–30) and the third is integrated databases (e.g. IC4R, Oryzabase and Gramene) (31–33). However, numerous genomic information of the species related to cultivated rice is still waiting to be incorporated into databases.

In this work, we constructed a new and user-friendly database termed RiceRelativesGD (http://ibi.zju.edu.cn/ricerelativesgd/), with the aim to serve it as a comprehensive genomic resource of rice relatives useful for rice breeding. We firstly integrated publicly available genomic resources from 2 cultivated rice and 11 rice relatives and identified 208 321 specific genes. We also identified valuable genomic resource for rice research, such as stress-related genes and photosynthesis genes, which is key to breed rice varieties with stress resistant and high efficiency of photosynthesis. Finally, practical bioinformatics online tools were provided to allow researchers to analyze genes with potential value for rice community.

## Materials and methods

### Data collection, classification and annotation

The current database included genomic datasets from 13 rice relatives that are publically available (Table 1). From the raw protein files of 13 genomes, the longest protein of each orthologous gene termed as ‘primary protein’ was extracted for gene family clustering analysis. Based on the Markov Cluster algorithm, 34 570 gene families were identified using Orthofinder v2.2.7 with sequence search program ‘diamond’ (34). And according to the gene family clustering results, genes present in multiple species were defined as ‘multi-species family genes’, genes present only in a single species were defined as ‘species-specific family genes’, while genes that could not be clustered to any gene family were defined as ‘orphan genes’. Species-specific family genes, orphan genes and multiple-species family genes that could not be clustered with *O. sativa* genes in the gene family analysis were further defined as ‘specific genes’ or, in other words, specific genes in RiceRelativesGD refer to genes without paralogs in *O. sativa* (*japonica* group) or *O. sativa* (*indica* group).

**Table 1:** List of sequenced genomes of rice and its relatives

Species	Genome types	Genome size	Predicted gene number	Accession	Reference
		(Estimated/assembly)			
*O. sativa (japonica* group*)*	AA (2*n* = 24)	420 Mb/390 Mb	35 825	Nipponbare	(15)
*O. sativa (indica* group)	AA(2*n* = 24)	−−/396 Mb	38 729	R498	(19)
*O. rufipogon*	AA(2*n* = 24)	−−/338 Mb	37 071	W1943	(20)
*O. nivara*	AA(2*n* = 24)	−−/338 Mb	36 313	IRGC100897	(20)
*O. glaberrima*	AA(2*n* = 24)	−−/316 Mb	33 164	CG14	(17)
*O. barthii*	AA(2*n* = 24)	−−/308 Mb	34 575	IRGC105608	(20)
*O. glumaepatula*	AA(2*n* = 24)	−−/373 Mb	38 149	GEN1233_2	(20)
*O. meridionalis*	AA(2*n* = 24)	−−/336 Mb	34 897	W2112	(20)
*O. puntaca*	BB(2*n* = 24)	−−/394 Mb	31 762	IRGC105690	(20)
*O. brachyantha*	FF(2*n* = 24)	297 Mb/261 Mb	32 037	IRGC101232	(16)
*Leersia perrieri*	−−/(2*n* = 24)	−−/267 Mb	29 078	IRGC105164	(20)
*Zizania latifolia*	−−/(2*n* = 34)	594 Mb/604 Mb	43 703	HSD2	(18)
*Echinochloa crus-galli*	−−/(2*n* = 6*x* = 54)	1.40Gb/1.27Gb	108 771	STB08	(7)

**Figure 1:**
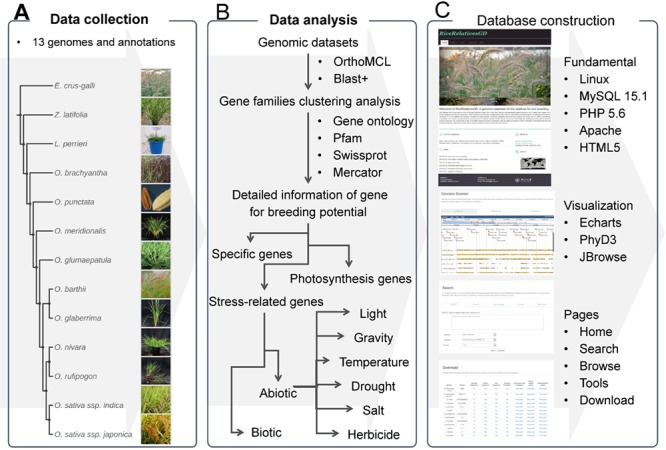
The flow diagram to show design and construction of RiceRelativeGD. (A) Data collection: genomic data from the cultivated rice and its relatives. (B) Data analysis: a flow chart to show the analyses used in the curation of the data included in the database, including the identification of specific genes and genes related to photosynthesis and stress tolerance. (C) Database construction: tools used in construction of the database and building functional modules of the database.

Detailed information on each gene, including organisms of origin, genomic location, family ID, gene structure, function descriptions and sequences, was displayed on the website. We used two approaches to annotate the exact function of each gene and its family. First, the primary protein sequences from 13 species were annotated with Pfam domain and GO terms using InterProscan v5.24–63.0 (35) and Mercator v4.0 with the default settings (36). Particularly, to better annotate gene functions of the genes included in RiceRelativesGD, we collected and used GO annotations and functional description of *O. sativa* (*japonica* group) from Rap-DB and MSU-RGAP. Second, we aligned the primary protein sequences to Swiss-prot dataset from Uniport (37) using ncbi-blast+ 2.6.0 with ‘-evalue 1e-5’ (38) and the best hit was extracted. The annotation results from both methods were integrated into our database. For each group of specific genes from different rice relatives, GOATOOLS (39) was applied to perform GO enrichment analysis with the GO annotation from InterProscan.

### Phylogenetic tree and genomic presence and absence variations

Based on the paralogs and orthologs identified by Orthofinder v2.2.7 (34), single-copy genes were identified and their primary protein sequences were aligned using MAFFT v7.310 (40). The tree was built using FastTree 2.1.7 with the default setting (41) and was drawn with ITOL (42).

To display genomic PAV in the database, genomes of the 11 rice relatives were aligned to *japonica* or *indica* rice genome, using the Nucmer program in the MUMer 3.23 package with the default setting (43); the alignment results were further filtered using the delta-filter program with parameter ‘-q’ and transformed into human readable format using the show-coords program with parameter ‘-lrTH’ in the MUMer 3.23 package (43). Finally, gff3 files were generated from the obtained results and visualized in the genome browser.

### Implementation of RiceRelativesGD

This database was built as a web-based system consisting of two major parts: one is data storage and management, and the other is high-level web interfaces displaying visualization functions (Figure 1). The backend of this system was constructed with MySQL 15.1, Perl 5.16 and PHP 5.6. HTML5, and JavaScript operating on the Apache web server was used to construct web interfaces. For the implementation of online tools in the database, several bioinformatics tools and external databases were integrated in this system. In the ‘search’ module, BLAST+ 2.2.29 (38) was used to perform sequence search. In the ‘Genome browse’ module, JBrowse 1.13.1 (44) was used to visualize genomic PAV (presence and absence variations) between cultivated rice and its relatives and specific genes. In the ‘build phyogenetic tree’ module, Mafft v7.310 (40), FastTree 2.1.7 (41) and PhyD3 (45) were used to perform sequence alignment, phylogenetic tree building and visualization.

**Figure 2:**
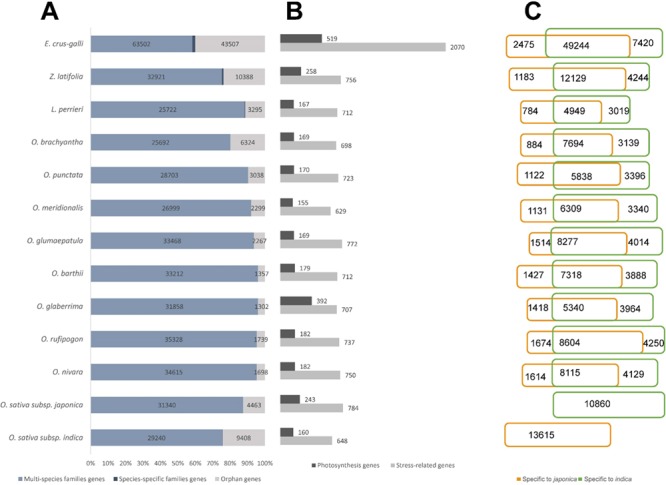
Statistics of the data included in RiceRelativeGD. (A) Stacked bar diagram to show the proportion of the three types of genes in each species. (B) Bar diagram to show the number of stress-related genes and photosynthesis genes in each species. (C) Venn diagram to show the number of genes specific to *japonica* or *indica* rice.

**Figure 3:**
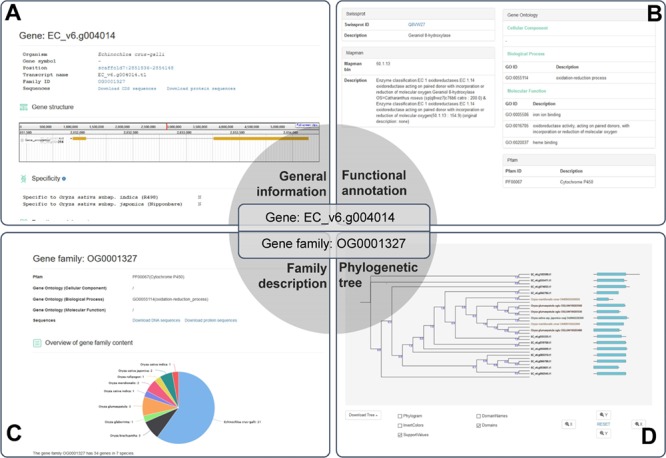
A case study for the application of RiceRelativesGD (showing the related information of the *EC_V6.g004014* gene). (A) General information about the gene. (B) Functional annotation of the gene. (C) Description of the gene family to which *EC_V6.g004014* belongs. (D) Phylogenetic tree of the genes in the gene family to which *EC_V6.g004014* belongs.

## Results

### The overall design of RiceRelativesGD

The overall design of RiceRelativesGD is shown in Figure 1. To better help users browse the data in the RiceRelativesGD, we set up four sections in the ‘Browse module’, including ‘Specific genes’, ‘Gene families’, ‘Stress-related genes’ and ‘Photosynthesis genes’. In the ‘Specific genes’ section, users can browse genes specific to *indica* rice (R498) or *japonica* rice (Nipponbare) by species (Figure 1). In the interface of each species, detailed information about the gene, such as gene family entry, statistics results and GO enrichment results, are shown. In the ‘Stress-related genes’ and ‘Photosynthesis genes’ sections, genes are further classified by their sub-classifications. For example, in the ‘Herbicide’ of ‘Stress-related genes’ sections, genes are further listed in four categories, including Cytochrome P450 monooxygenases, Glutathione S-transferases, ABC transporters and Glycosyltransferases. All these datasets can be downloaded in the ‘Download’ module along with the statistics of the gene of interest or in each species.

RiceRelativesGD provides three online tools for the users: (1) the ‘Search’ tool performing sequence and keyword (gene name, GO term, Pfam and Swissprot term) search; (2) the ‘Genome browse’ tool providing a genome browser for each species. In the genome browser interfaces of ‘*indica* rice’ and ‘*japonica* rice’, genomic PAV of each rice relative and genes specific to *indica* or *japonica* rice can be observed, (3) the ‘Build phylogenetic tree’ tool providing an online tool for phylogenetic analysis. Users can input the gene names from the RiceRelativesGD database or paste sequences from external sources to construct a phylogenetic tree (Figure 1).

### The overall analysis results

RiceRelativesGD incorporates publicly available genomic information from 12 species of the *Oryzoideae* subfamily (13,16–18,20,46) and the paddy weed *E. crus-galli* (7). A total of 34 570 gene families were identified, including 34 147 gene families with members from multiple species (multi-species families) and 423 gene families with members from only one species (species-specific families). Additionally, 91 085 orphan genes were found (Figure 2A). From the 11 rice relatives, a total of 208 321 genes were found to be specific to *indica* or *japonica* rice genome **(**Figure 2C).

Besides, the database also provides potentially valuable genes for rice breeding. We identified a total of 13 643 genes related to external stimulus responses (light, gravity, temperature, drought, salinity and abiotic stress) or photosynthesis (photophosphorylation, Calvin cycle, photorespiration and C4 photosynthesis) from the rice relatives **(**Figure 2B**)**. The stress-related genes from rice relatives provide potential breeding targets for enhancing stress resistance of rice. Genes identified to be involved in the C4 photosynthesis pathways could be useful targets for improving the efficiency of rice production with less input of chemical fertilizers.

### A case study for the application of RiceRelativesGD

The database provides detailed information for each gene, including organism of origin, genomic location, family ID, gene structure, function annotations and sequences. As an example *EC_v6.g004014* is shown in Figure 3, which was defined as a gene encoding Cytochrome P450 based on Pfam domain, Swissprot and Mercator analyses. *EC_v6.g004014* belongs to the OG0001327 gene family, which has 34 genes from 7 species. In the interface of the gene family OG0001327, users can find function annotations, overview of the gene family and the phylogenetic tree of all genes of the family as well as download sequences. The phylogenetic tree of the gene family includes not only family members but also the Pfam domain information of each gene allowing researchers to easily observe the differences and similarities among the family members. The phylogenetic tree can be downloaded as image or tree file by clicking ‘Download Tree’ **(**Figure 3).

## Discussion

Rice relatives have become increasingly important for future improvement of rice varieties as they retain many competitive agronomic traits that have lost in rice during domestication and breeding with intensive artificial selection. Re-introducing these genetic elements back into the rice genetic background would not only enhance the performance of rice but also alleviate the increased genetic load caused by domestication and breeding (47). Genomic data of rice relatives are essential and crucial sources for uncovering the genes lost in the cultivated rice. Currently, most rice genomic databases do not provide information on rice relatives. Even though a few databases (e.g. Gramene, Ensemble Plants or PLAZA) integrate genomic resources of some rice relatives, they mainly focus on providing general information on the genomes, such as their orthologs and paralogs, gene gain/loss tree and genomic alignments, and pay no attention to a particular group of rice relatives or analysis of specific genes in rice relatives that could be valuable for modern rice breeding programs. RiceRelativesGD fills the gap by providing not only more comprehensive genomic information of rice relatives for the rice community but also specific genes from rice relatives, including stress-related genes, photosynthesis genes and so on. RiceRelativesGD collected and organized published genomic data of rice and its relatives from relevant literatures. Currently, a total of 208 321 specific genes from rice relatives are deposited in RiceRelativesGD.

In RiceRelativesGD, disagreement can be found between phylogenetic relationship (Fig. 1A) and specific gene number. Different genome assembly and gene prediction categories applied in each genome could affect the specific gene numbers. However, the difference of gene numbers or specific gene numbers may not be a convincing evidence to measure the phylogenetic distance among species. The protein-coding gene numbers were dynamically changed when the duplication, divergence or recombination events happened. For example, approximately 50 *de novo* genes generated and retained in recent divergence of *Oryza* per million years was validated (48). With the rapid development of sequencing technology and the declining sequencing cost, an increasing number of more complete genomes of rice relatives will be available, which could eliminate this disharmony. To keep pace with this trend, RiceRelativesGD should and will be updated to include all published genomic data of rice relatives. RiceRelativesGD is the first database focusing on providing comprehensive information on specific genes of diverse rice relatives. It will facilitate researchers mining genes with potential value for modern rice breeding. We will not only regularly update RiceRelativesGD with newly published data but also enhance the functionality with the aim of serving it as a foundation for future studies on rice relatives.

## Notes

**Database URL:** http://ibi.zju.edu.cn/ricerelativesgd/
